# The Use of Velocity Information in Movement Reproduction

**DOI:** 10.3389/fpsyg.2017.00983

**Published:** 2017-06-12

**Authors:** Sergio Chieffi, Antonietta Messina, Ines Villano, Anna A. Valenzano, Ersilia Nigro, Marco La Marra, Giuseppe Cibelli, Vincenzo Monda, Monica Salerno, Domenico Tafuri, Marco Carotenuto, Luigi Cipolloni, Maria P. Mollica, Marcellino Monda, Giovanni Messina

**Affiliations:** ^1^Department of Experimental Medicine, University of Campania “Luigi Vanvitelli”Naples, Italy; ^2^Department of Clinical and Experimental Medicine, University of FoggiaFoggia, Italy; ^3^CEINGE-Biotecnologie Avanzate ScarlNapoli, Italy; ^4^Department of Motor Sciences and Wellness, University of Naples “Parthenope”Naples, Italy; ^5^Center for Childhood Headache, Department of Mental Health, Physical and Preventive Medicine, Clinic of Child and Adolescent Neuropsychiatry, Università degli Studi della Campania “Luigi Vanvitelli”Naples, Italy; ^6^Università degli Studi di Roma La SapienzaRome, Italy; ^7^Department of Biology, Università degli Studi di Napoli Federico IINaples, Italy

**Keywords:** proprioception, memory, velocity, arm movement, humans

## Abstract

**Background:** Previous studies suggested that movement velocity influence space perception.

**Aim and Objectives:** We examined whether healthy participants used velocity information when they were asked to reproduce a previously performed movement. Two experiments were carried out.

**Methods:** In Experiment 1, blindfolded participants actively performed an arm movement (criterion movement, CM) at a natural velocity, or quickly, or slowly. After a brief delay, participants were asked to reproduce (reproduction movement, RM) CM-amplitude. No velocity constraints were imposed in making RM. In Experiment 2, CM was performed quickly or slowly. After a brief delay, the participants were asked to reproduce not only CM-amplitude but also CM-velocity.

**Results:** Experiment 1: in Natural condition, RM-velocity did not differ from CM-velocity and the participants accurately reproduced CM-amplitude. Conversely, in Fast and Slow condition, RM-velocities differed from CM-velocities and in Slow condition RM-amplitude was greater than CM-amplitude. Experiment 2: both RM-amplitude and -velocity did not differ from CM-amplitude and -velocity.

**Conclusion:** The present study confirms the view that movement velocity influences selectively space perception and suggests that this influence is stronger for slow than fast movements. Furthermore, although velocity information is crucial in accurately reproducing CM-amplitude, it was not used spontaneously when movements were performed at unnatural velocities.

## Introduction

When subjects are asked to perform an arm movement (criterion movement, CM) and then to reproduce it (reproduction movement, RM), both motor program and sensory cues related to CM can be stored in working memory and used in making RM (Poulton, 1981). If vision is excluded, individuals rely on proprioceptive information about limb position and movement (Clark and Horch, 1986; Gandevia et al., 1992; Chieffi et al., 2004, 2017; Iavarone et al., 2007; Proske and Gandevia, 2012) in egocentric frames of reference (Chieffi et al., 2009, 2012, 2014; Chieffi, 2016). It is generally accepted that muscle receptors are the main contributors of perception of arm position and movement (McCloskey, 1973, 1978; Hollins and Goble, 1988), while cutaneous and joint receptors appear to play only a subsidiary role (Ferrell et al., 1987). There are two main types of muscle receptors, namely the primary (group Ia) and secondary (group II) sensory endings in muscle spindles. The secondary endings provide position information related to the length of the muscle (McCloskey, 1973, 1978; Matthews, 1988; Gandevia et al., 1992). The response of primary endings varies with both the length of the muscle and the rapidity of stretch and provide relatively more information on limb velocity (McCloskey, 1973, 1978; Matthews, 1988; Gandevia et al., 1992). Both position and velocity information about CM may be stored in working memory and used to perform RM. Position signals might yield information about both the final arm configuration and the space covered by CM as the difference between final and initial arm position (CM-amplitude). Velocity signals, on the other hand, might provide information about the spatio-temporal properties of CM.

Interestingly, previous experiments showed that perception of movement amplitude could be influenced by the velocity with which the movement itself was performed. The amplitude of slow movements was overestimated and that of fast movements was underestimated (Hollins and Goble, 1988; Chieffi et al., 2004). Hollins and Goble (1988) asked to the blindfolded subjects to move their index finger until a stop was encountered and to give a magnitude estimate of the distance traversed. The results showed that magnitude estimates of distance increased as a function of actual distance, but decreased as a function of movement velocity (Hollins and Goble, 1988). Furthermore, Chieffi et al. (2004) found that movement velocity influenced the kinaesthetic coding of target location in short-term memory. The authors (Chieffi et al., 2004) required the blindfolded subjects to reach, with RM, the remembered CM-end point. Subjects made overshoot errors when the CM was performed slowly and the RM quickly and undershoot errors when the CM was performed quickly and the RM slowly (Chieffi et al., 2004).

Given that movement velocity affects space perception, it is possible to hypothesize that the use of CM-velocity information is necessary to accurately reproduce CM-amplitude. Then, an interesting question is to examine whether participants use spontaneously CM-velocity information when they are asked to reproduce CM-amplitude. To answer this question we carried out the Experiment 1.

## Experiment 1

In the present experiment blindfolded participants performed actively CM at a natural velocity, or slowly, or quickly. After a short delay, they were asked to reproduce CM-amplitude. No velocity constraints were imposed in making RM. Our hypothesis was as follows. If participants used CM-velocity information in reproducing CM-amplitude, they would have accurately reproduced CM-amplitude. Conversely, if participants did not use CM-velocity information, they would have failed to accurately reproduce CM-amplitude.

### Methods

Twenty healthy and right-handed subjects (12 females and 8 males; mean age 23.8 years; *SD* = 3.8) participated in the experiment. The participants were naive to the task. The experiment was approved by the ethics committee of the “Azienda Ospedaliera Universitaria, Seconda Università di Napoli” and was performed in accordance with the 1964 Declaration of Helsinki. Participants gave written informed consent to take part in the study.

The participants were blindfolded and sat in front of a table on which a digitizing tablet was placed. The tablet measured 430 mm (width) × 570 mm (depth) and had an active surface of 305 × 458 mm. It was contacted with a non-inking electronic stylus. When in contact, the position of the stylus tip was sampled at a rate of 50 Hz. Data were recorded in horizontal and vertical coordinates with an accuracy of 0.25 mm. The tablet was covered with a thin white card on which the starting position was drawn in black ink (a 3 mm diameter spot) at 16 cm from the trunk along the mid-sagittal axis. Two horizontal lines were also drawn 25 and 40 cm from the starting position. These lines delimited the working space.

The participants held the stylus with their right hand. In the training phase, the experimenter placed the participant’s hand on the starting position and passively moved it up to the lines in order to make the participant aware of the extent of working space. Then, the participant was required to perform movements at a natural velocity along the mid-sagittal axis and stop in the working space. At the end of each movement, the experimenter brought the participant’s hand back to the starting position. The experimental session started once the participant reached correctly the working space with three consecutive movements. At this time, the participant was required to reach the working space with a movement (criterion movement, CM) performed at natural velocity, or “very slowly,” or “very quickly” (respectively Natural, Slow and Fast condition). As soon as the participant ended the CM, the experimenter brought the participant’s hand back to the starting position. After 2 s, the participant was asked to reproduce (reproduction movement, RM) CM-amplitude. There were no constraints on RM-velocity, i.e., the participant was free to use the velocity she/he preferred. There were a total of 30 experimental trials (3 velocity conditions × 10 movements). The trials were divided into two blocks. In the first block, CMs were performed at natural velocity. In the second block, slow and fast CMs were performed randomly.

Movement amplitude, time and mean velocity were measured. Movement amplitude was calculated using the following formula

$$\begin{array}{c} \surd ({\left(x_{2}-x_{1}\right)}^{2}+{\left(y_{2}-y_{1}\right)}^{2}). \end{array}$$

Separate ANOVAs were conducted on mean values of movement amplitude, time and mean velocity with Velocity (Natural, Slow and Fast) and Movement (CM, RM) as the within-subjects’ factors. Paired comparisons were performed using Bonferroni *post hoc* test. Furthermore, within-subject correlation coefficients (Spearman’s rho) between RM- and CM-velocity were calculated for each velocity condition.

Significance level was fixed at *p* < 0.05.

### Results and Discussion

**FIGURE 1:**
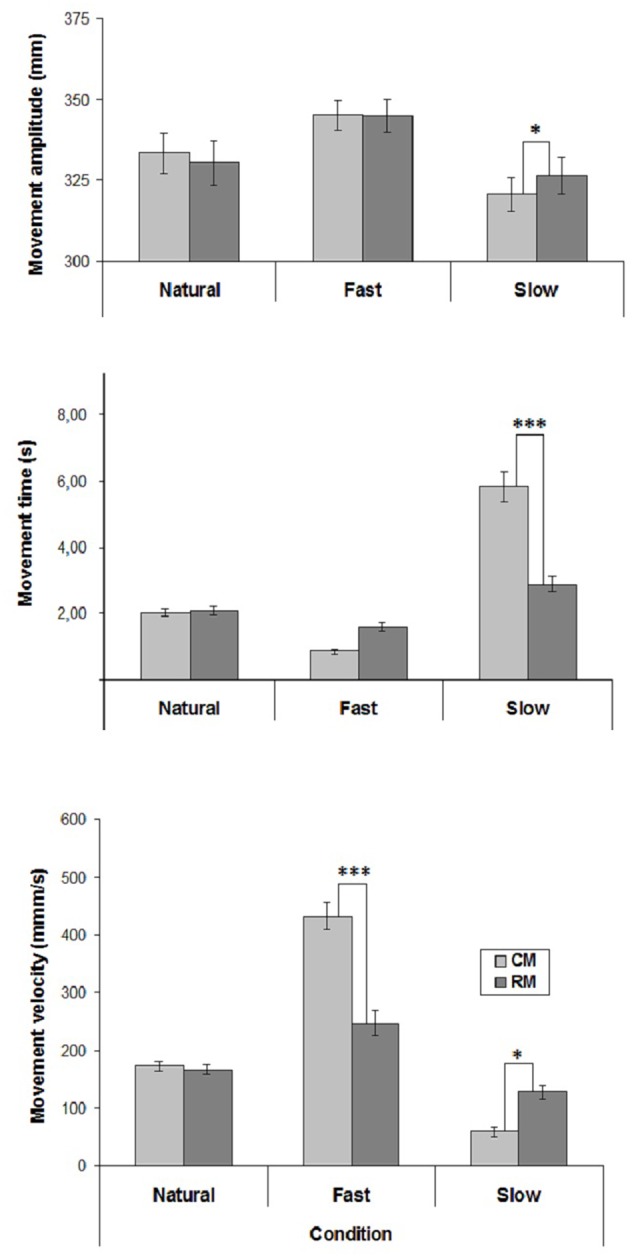
Mean values of movement amplitude, time and mean velocity in the three experimental conditions of Experiment 1. Mean values are shown with SE (*bars*). CM, criterion movement; RM, reproduction movement. ^∗^*p* < 0.01, ^∗∗^*p* < 0.001, ^∗∗∗^*p* < 0.0001.

The mean values of CM- and RM- amplitude, time and velocity are represented in **Figure 1**.

Velocity influenced movement amplitude [*F*(2,38) = 10.16, *p* < 0.001] and there was a significant interaction between Velocity and Movement [*F*(2,38) = 8.84, *p* < 0.001]. *Post hoc* comparisons showed that in both Natural and Fast conditions RM-amplitude did not differ from CM-amplitude. Conversely, in Slow condition RM-amplitude was greater than CM-amplitude (*p* < 0.01). Furthermore, Fast CM-amplitude was greater than Natural and Slow CM-amplitudes (*p* < 0.0001), and Natural CM-amplitude was greater than Slow CM-amplitude (*p* < 0.001).

Both Velocity [*F*(2,38) = 82.43, *p* < 0.00001] and Movement [*F*(1,19) = 18.61, *p* < 0.001] influenced movement time. Furthermore, Velocity significantly interacted with Movement [*F*(2,38) = 43.08, *p* < 0.00001]. *Post hoc* comparisons showed that in Natural and Fast conditions RM-time did not differ from CM-time, whereas in Slow condition RM-time was lesser than CM-time (*p* < 0.0001). Furthermore, Natural RM-time did not differ from Fast and Slow RM-times, whereas Fast RM-time was greater than Slow RM-time (*p* < 0.002).

Both Velocity [*F*(2,38) = 105.42, *p* < 0.0001] and Movement [*F*(1,19) = 17.49, *p* < 0.001] influenced movement velocity. There was also a significant interaction between Velocity and Movement [*F*(2,38) = 53.91, *p* < 0.0001]. *Post hoc* comparisons showed that whereas in Natural condition RM-velocity did not differ from CM-velocity, in Fast condition RM-velocity was lesser than CM-velocity (*p* < 0.0001), and in Slow condition RM-velocity was greater than CM-velocity (*p* < 0.01). Furthermore, Fast RM-velocity was greater than Natural and Slow RM-velocities (*p* < 0.001), and Slow RM-velocity did not differ Natural RM-velocity.

Regarding the correlation coefficients between CM- and RM- velocities calculated individually for each subject, a significant positive correlation was found in Natural condition in 16 cases out of 20, and only in 3 cases out 20 in both unnatural conditions (mean correlation coefficients: Natural condition, *r* = 0.68; Slow condition, *r* = 0.22; Fast condition, *r* = 0.29).

The results of the present experiment showed that in the Natural condition RM-velocity did not differ from CM-velocity, and the participants accurately reproduced CM-amplitude. Conversely, in both unnatural velocity conditions, RM-velocity differed from CM-velocity, and in the Slow condition the participants overestimated CM-amplitude. These observations suggest that the reproduction of CM-velocity is an important requirement to accurately reproduce CM-amplitude.

## Experiment 2

The results of the previous experiment suggest that although CM-velocity reproduction was crucial to accurately reproduce CM-amplitude, this occurred only in Natural condition. In the present experiment we examined whether blindfolded participants were able to reproduce explicitly fast and slow CM-velocities or not.

### Methods

Sixteen subjects (11 females and 5 males; mean age 22.4 years; *SD* = 3.5) who took part in the Experiment 1 participated to the Experiment 2. They performed the experiment at least a week after taking part in the experiment 1. The experiment was approved by the ethics committee of the “Azienda Ospedaliera Universitaria, Seconda Università di Napoli” and was performed in accordance with the 1964 Declaration of Helsinki. Participants gave written informed consent to take part in the study.

The apparatus was the same as Experiment 1. Training phase was restricted to slow and fast velocities. In the experimental session, the participant was required to reach the working space with a movement (CM) performed “very slowly” or “very quickly.” As soon as the subject ended the CM, the experimenter brought the subject’s hand back to the starting position. After 2 s, the subject was asked to reproduce both CM-velocity and amplitude. There were a total of 20 experimental trials (2 velocity conditions × 10 movements). CM-velocities were performed randomly.

Separate ANOVAs were conducted on mean values of movement amplitude, time and mean velocity, with Velocity (Slow, Fast) and Movement (CM, RM) as the within-subjects’ factors. Paired comparisons were performed using Bonferroni *post hoc* test. Furthermore, within-subject correlation coefficients (Spearman’s rho) between RM- and CM-velocity were calculated for each velocity condition. Significance level was fixed at *p* < 0.05.

### Results and Discussion

**FIGURE 2:**
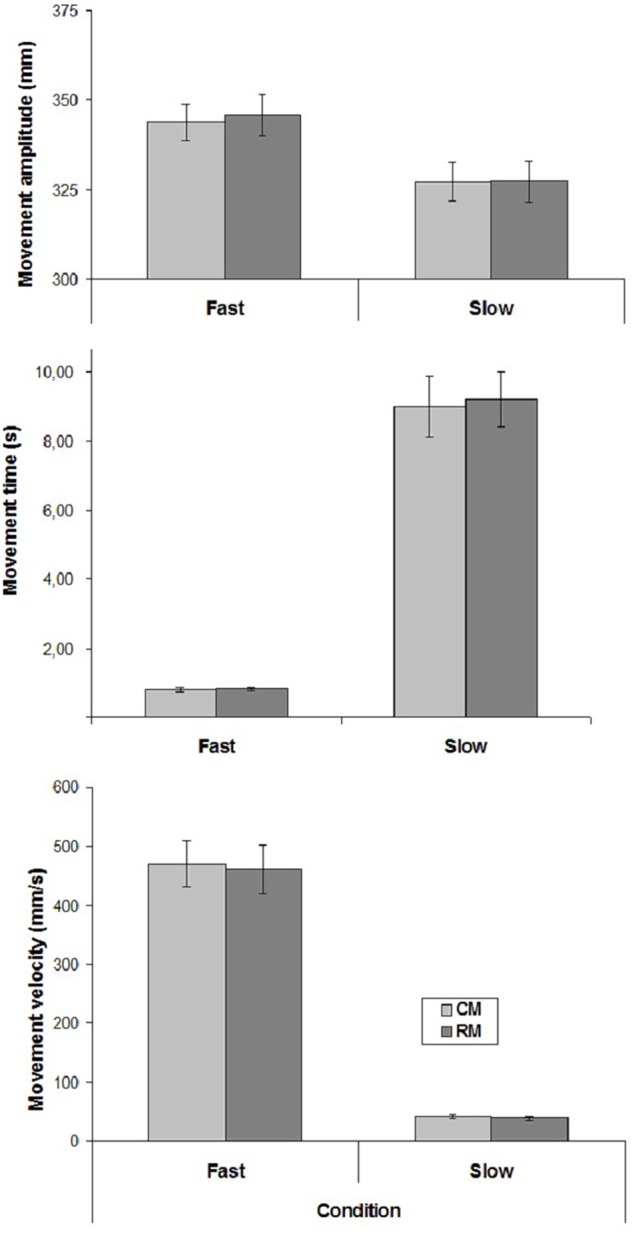
Mean values of movement amplitude and mean velocity in the two experimental conditions of Experiment 2. Mean values are shown with SE (*bars*). CM, criterion movement; RM, reproduction movement.

The mean values of CM- and RM- amplitude, time and velocity are represented in **Figure 2**.

Only Velocity influenced movement amplitude, time and velocity [amplitude: (F(1,15) = 10.24, *p* < 0.01; time: *F*(1,15) = 101.34, *p* < 0.00001; velocity: F(1,15) = 113.16, *p* < 0.00001)]. There was no significant effect of Movement, and no interaction.

Regarding the correlation coefficients between CM- and RM- velocities, a significant positive correlation was found in 11 cases out of 16 in both conditions (mean correlation coefficients, Slow condition: *r* = 0.57; Fast condition: *r* = 0.60).

Taken together, the results of the present experiment suggest that the participants were able to reproduce slow and fast velocities when explicitly requested.

## General Discussion

The main findings of the present study were: (i) the influence of movement velocity on space perception was stronger for slow than fast movements. In other words, for slow movements minor variations in velocity produced greater changes in space perception; (ii) although velocity information was relevant in reproducing the CM-amplitude it was not used spontaneously when the movements were performed at unnatural velocities.

Regarding the influence of movement velocity on perception of the distance traversed, this phenomenon was reported in previous studies (Hollins and Goble, 1988; Chieffi et al., 2004). These studies showed that the amplitude of slow movements was overestimated and that of fast movements underestimated. The results of the present study were in agreement with this view. We observed that both in the Experiments 1 and 2 CM-amplitude varied as a function of CM-velocity. In Experiment 1 Fast CM-amplitude was greater than Natural and Slow CM-amplitudes, and Natural CM-amplitude was greater than Slow CM-amplitude. Similarly, in the Experiment 2 Fast CM-amplitude was greater than Slow CM-amplitude. It is worth noting that in our experiment the subjects were free to choose the CM-amplitude, and the choice of standard amplitude should have been an economical strategy to perform the task. However, this did not occur. One possible interpretation of this movement effect is that space representation depended on incoming movement sensory input so that the space covered slowly was represented longer than that covered rapidly. In the present study, fast movements lasted on average 0.80 s. and slow movements 7.40 s. This movement time was sufficient for processing incoming sensory input as on-line corrections based on proprioceptive feedback have been observed to take place in 50–100 ms (Lee and Tatton, 1975; Crago et al., 1976; Smeets et al., 1990). Furthermore, other researchers reported that error detection capability was more effective in very quick movements (i.e., 210 ms) than slower movements (350 ms) (Clark et al., 1985; Lönn et al., 2001; Kerr and Worringham, 2002; Sherwood, 2010).

It is plausible that the effect of movement velocity on space perception produced the CM-amplitude reproduction error found in the Slow condition of the Experiment 1. In this condition RM-velocity was higher than CM-velocity. It is possible that the subjects perceived the space traversed during RM shorter than that perceived during CM so that they stopped the RM farther than the true CM end-point. Interestingly, in the Fast condition of the Experiment 1 CM-amplitude was accurately reproduced. In this condition RM-velocity was lower than CM-velocity. Note that the difference in absolute value between the CM- and RM-velocity was about 67 mm/s in Slow condition and 184 mm/s in Fast condition. This observation suggests that the influence of movement velocity on perception of space covered was stronger for movements performed slowly than for those executed quickly. An alternative explanation of this finding is that the firing pattern of primary endings was used to evaluate movement duration and then movement amplitude (Hollins and Goble, 1988). Hollins and Goble (1988) proposed that the movement duration, obtained from firing pattern of primary endings, might be a valuable indicator of the extent of a movement only for those movements carried out within a narrow and stable range of velocities (Paillard and Brouchon, 1974; Hollins and Goble, 1988). This range would vary with the nature of movement and be somewhat different in the different individuals. However, for a particular person and experimental situation, there is a clear and consistently employed preferred velocity. Thus, given this consistency in the movement velocity, movement amplitude might be obtained by registering his duration, as indicated by the period of increased firing of primary endings in muscle spindles (Paillard and Brouchon, 1974; Hollins and Goble, 1988). Conversely, if the movement is performed at a different velocity, the information derived from primary endings should be inaccurate so that the extent of slow movements would be overestimated and that of fast movements underestimated (Hollins and Goble, 1988). Furthermore, it is interesting to note that in our experiment the difference in absolute value between RM- and CM-time in Slow condition was greater than that in Fast condition (respectively, 2.95 and 0.73 s). This greater difference in time duration might have produced, in Slow condition, a significant difference in perception of movement extent between CM and RM.

The presence of an error in the reproduction of CM-amplitude suggests that participants did not spontaneously use velocity information, although the latter was crucial to accurately reproduce movement amplitude. Only in Natural condition participants reproduced CM-velocity as well as CM-amplitude. Conversely, they did not reproduce CM-velocity in unnatural velocity conditions. In the Experiment 2, we explored the possibility that the participants did not spontaneously use velocity information because they were not able to reproduce fast or slow velocities. This hypothesis was not supported by the results of the Experiment 2 showing that the participants accurately reproduced fast and slow movement velocities when explicitly requested.

## Conclusion

The data of the present study confirmed the view that movement velocity influences space perception and suggested that this influence is stronger for slow than fast movements. Furthermore, although velocity information is crucial in accurately reproducing CM-amplitude, it was not used spontaneously when the movement was performed at an unnatural velocity.

## Author Contributions

SC, AM, GC, VM, and MPM carried out the study; IV, AV, EN, MS, MLM, LC, DT participated in the design of the study; SC, MC, MM, and GM participated in the design and coordination and helped to draft the manuscript. All authors read and approved the final manuscript.

## Conflict of Interest Statement

The authors declare that the research was conducted in the absence of any commercial or financial relationships that could be construed as a potential conflict of interest.
